# Neural Evidence of the Cerebellum as a State Predictor

**DOI:** 10.1007/s12311-018-0996-4

**Published:** 2019-01-09

**Authors:** Hirokazu Tanaka, Takahiro Ishikawa, Shinji Kakei

**Affiliations:** 1 School of Information Science, Japan Advanced Institute of Science and Technology, 1-1 Asahidai, Nomi, Ishikawa 923-1211 Japan; 2 Movement Disorders Project, Tokyo Metropolitan Institute of Medical Science, 2-1-6 Kamikitazawa, Setagaya, Tokyo, 156-8506 Japan

**Keywords:** Motor control, Internal forward model, Mossy fiber, Purkinje cell, Dentate cell, Kalman filter

## Abstract

We here provide neural evidence that the cerebellar circuit can predict future inputs from present outputs, a hallmark of an internal forward model. Recent computational studies hypothesize that the cerebellum performs state prediction known as a forward model. To test the forward-model hypothesis, we analyzed activities of 94 mossy fibers (inputs to the cerebellar cortex), 83 Purkinje cells (output from the cerebellar cortex to dentate nucleus), and 73 dentate nucleus cells (cerebellar output) in the cerebro-cerebellum, all recorded from a monkey performing step-tracking movements of the right wrist. We found that the firing rates of one population could be reconstructed as a weighted linear sum of those of preceding populations. We then went on to investigate if the current outputs of the cerebellum (dentate cells) could predict the future inputs of the cerebellum (mossy fibers). The firing rates of mossy fibers at time *t* + *t*_1_ could be well reconstructed from as a weighted sum of firing rates of dentate cells at time *t*, thereby proving that the dentate activities contained predictive information about the future inputs. The average goodness-of-fit (*R*^2^) decreased moderately from 0.89 to 0.86 when *t*_1_ was increased from 20 to 100 ms, hence indicating that the prediction is able to compensate the latency of sensory feedback. The linear equations derived from the firing rates resembled those of a predictor known as Kalman filter composed of prediction and filtering steps. In summary, our analysis of cerebellar activities supports the forward-model hypothesis of the cerebellum.

## Introduction

The cerebellum plays a critical role in the control and coordination of body movements, adaptation to novel environments, and acquisition of new motor skills. Evidence from clinical observations and psychophysical experiments indicates that impairments of the cerebellum lead to motor ataxia characterized by incoordination and dysmetria in multijoint movements. Clinical evidence pioneered by the seminal work of Holmes suggests that impairments in the cerebellum could lead to symptoms characterized by lack of coordination across multiple degrees of freedom in motor control, collectively known as cerebellar ataxia [1]. The cerebellum also plays an essential role both in adapting to external perturbations such as visual rotation or external force fields and in acquiring a new skill [2–6]. The cerebellum is one of the central nervous system whose anatomical structure, cytoarchitecture, and electrophysiological properties have been thoroughly studied. Despite the plethora of clinical and psychophysical evidence, the precise mechanisms by which the cerebellum coordinates body movements are not yet understood.

Recent computational studies suggest that the cerebellum predicts current and future states of the body by solving the dynamics with given efference copy of motor commands, known as the computation of an internal forward model [7–10]. Sensory feedback signals from the periphery have certain delays in reaching the central nervous system, on the orders of a few tens to 100 ms. Therefore, the brain always receives the “past” state of the body. It is known in engineering and mechanics that feedback control based on time-delayed state can behave in an oscillatory and often unstable way if the delay is on the order of time constants of system dynamics. Fortunately, physical laws that govern body movements allow the brain to predict a current state from a previous state and an efference copy of motor command, essentially by solving the Newtonian mechanics. This predictive mechanism allows stable and dexterous control of body movements. Although the cerebellum has been suggested as a locus of forward-model computation from psychophysical, neuroimaging, and stimulation studies [11–13], the neural mechanism of how the cerebellum performs predictive computation has not yet been understood [10].

We therefore set out to determine the computation in the cerebellar circuit in a monkey during wrist step-tracking movements and provide neural evidence that current outputs of the cerebellar circuit contain predictive information about future inputs, a hallmark of an internal forward model. We analyzed firing rates of mossy fibers (MFs) (inputs to the cerebellar cortex), Purkinje cells (PCs) (outputs from the cerebellar cortex to the dentate nucleus (DN)), and dentate cells (DCs) (output of the cerebellum) of a monkey performing step-tracking wrist movements. Unlike the cerebral cortex in which multiple inputs and multiple outputs are related in a highly complex way, the cerebellar MF input and DN output are well defined and basically organized in a characteristic feedforward manner. By exploiting the feedforward structure, firing rates of one population were reconstructed from those of other populations that innervate to the target population. Also, we investigated if firing rates of cerebellar output (i.e., DCs) at time *t* contained information that was predictive for future inputs to the cerebellar cortex (i.e., MFs) at time *t* + *t*_1_. Our analyses targeted to test the forward-model hypothesis of the cerebellum.

## Methods

### Behavioral Task and Electrophysiological Recording

We used firing rates of the cerebellar cells reported in our previous publications. Here, a brief description about the behavioral task and the electrophysiological recording is provided, and for a full account, please refer to the previous publications [14, 15]. All surgical and experimental protocols were approved by the Animal Care and Use Committee of Tokyo Metropolitan Institute of Medical Science. A male macaque monkey participated in a step-tracking movement task of the right wrist. The monkey gripped a manipulandum that measured the wrist movements and controlled a cursor on a computer screen with the manipulandum toward one of eight targets that were uniformly located on a circle of radius of 8°, corresponding to a wrist movement of 20°. An initial posture of the wrist was either pronated or supinated, so there were 16 experimental conditions (eight movement directions × two initial postures).

During the step-tracking movement task, neural activities of MFs, PCs, and DCs were recorded. Their anatomical locations and physiological characteristics allowed us to identify these cells with confidence. Spike data were sorted on the timing of movement onsets and binned into firing rates of a time window of 20 ms averaged over 10 to 20 trials for one condition. The dataset included 94 MFs, 83 PCs, and 73 DCs from monkey 1. PCs were identified by the coexistence of simple and complex spikes, and MFs were identified by the occurrence of a short positive–negative potential followed by a longer negative afterwave [14, 15]. DCs were identified, in addition to anatomical separation from the cerebellar cortex, by the characteristic of large negative–positive spike waveforms [14]. Note that only simple spikes were analyzed for PCs because the monkey had been trained for the task for years and no apparent improvement in task performance occurred during the recordings. These cells were recorded on different experimental sessions or days, so the firing rates but not spikes were analyzed in this study.

### Characterization of Firing Rates: Spatiotemporal Separability Index and Distributions

**Fig. 1:**
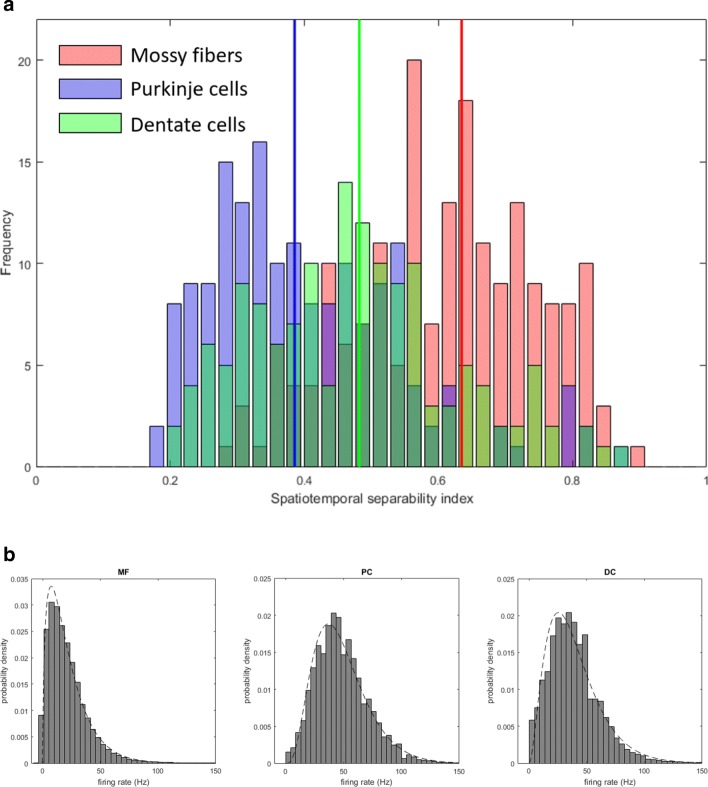
**a** Distribution of the spatiotemporal separability index (STSI) computed for mossy fibers (MFs) (red), Purkinje cells (PCs) (blue), and dentate cells (DCs) (green). Three colored vertical lines depict the median values of the three cell populations. **b** Probability densities of firing rates of MFs (left), PCs (middle), and DCs (right). The densities are binned into 50 bins. Dashed lines overlapped with the histograms represent best-fit Gamma distributions

We here examine the spatiotemporal pattern of activities of single neurons. Specifically, we introduce first an index of spatiotemporal separability and then examine its distributions for the different neuron groups. Let ${\mathrm{MF}}_i^{p,d}(t)$ denote the firing rates at *t*-th time bin in [− 1 s, + 1 s] recorded from *i*-th MF for direction *d* and posture *p*. Similarly, ${\mathrm{PC}}_i^{p,d}(t)$ and ${\mathrm{DC}}_i^{p,d}(t)$ are defined for PCs and DCs, respectively. We first characterize the spatiotemporal patterns of activities of MFs, PCs, and DCs and introduce an index that quantifies how spatiotemporally separable the activities are. If a cell has stationary directional tuning, the firing rates are spatiotemporally separable as a product of a function of movement direction and a function of time (Fig. 1a). If directional tuning of a cell is not stationary but rather exhibits time-varying preferred direction, the firing rates are spatiotemporally nonseparable. For a given posture, the firing rates of *i*-th neuron are summarized into a matrix form as **R** ∈ *ℝ*^*D* × *T*^, where *D* is the number of movement directions, *T* is the number of time windows of recording, and *D* ≪ *T*. To level off the difference in firing rates, each row of the matrix **R** is normalized to zero mean and unit variance. **R** may be decomposed into a factorial form as

1 $$ \mathbf{R}=\mathbf{U}\boldsymbol{\Sigma } {\mathbf{V}}^{\top }=\sum \limits_{d=1}^D{\sigma}_d{\mathbf{u}}_d{\mathbf{v}}_d^{\top } $$

Here ${\left\{{\sigma}_d\right\}}_{d=1}^D$ are singular values of a descending order, *σ*_1_ ≥ ⋯ ≥ *σ*_*D*_. {**u**_*d*_} and {**v**_*d*_} are *D*-dimensional and *T*-dimensional orthonormal vectors characterizing directional tuning and temporal profile of firing rates, respectively. If directional tuning is invariant during experimental duration, then **R** is expressed as a rank-one matrix (i.e., $\mathbf{R}={\sigma}_1{\mathbf{u}}_1{\mathbf{v}}_1^{\top }$). On the other hand, if directional tuning varies during movement duration, then **R** cannot be a rank-one matrix and contains multiple rank-one matrices. Therefore, how well **R** is reconstructed by a rank-one matrix is a candidate measure for characterizing properties of firing rates. Once the firing-rate matrix is decomposed into a sum of rank-one matrices, the degree of spatiotemporal separability is quantified by an index (spatiotemporal separability index, STSI),

2 $$ \mathrm{STSI}=\frac{\sigma_1^2}{\sum_{d=1}^D{\sigma}_d^2} $$

STSI takes a value ranging from 0 to 1; STSI is 1 if **R** is spatiotemporally separable, whereas STSI takes a smaller value if **R** contains multiple rank-one matrices. This index was computed for each cell.

To further characterize the firing rates, we also examined distributions of firing rates of the three populations. For each population, a histogram was constructed by counting the frequency of firing rates in all time bins of cells in that population both for pronated and supinated postures. These histograms were fitted with Gaussian, Gamma, Rayleigh, and inverse Gaussian distributions using a maximum likelihood method, and the values of Akaike information criterion (AIC) were computed for these distributions.

### Linear Reconstruction of Firing Rates of PCs

We then attempted to reconstruct the firing rate as a weighted sum of those of preceding layers by exploiting the connectivity of the cerebellum (Fig. 1 inset). Namely, PCs receive inputs from MFs through granule cells and inhibitory interneurons (stellate cells and basket cells), and that DCs receive inhibitory and excitatory inputs from PCs and collaterals of MFs, respectively.

An activity of *i*-th PC of time *t* was reconstructed as a weighted sum of those of MFs at the same time as

3 $$ {\widehat{\mathrm{PC}}}_i^{p,d}(t)=\sum \limits_{j=1}^{N_{\mathrm{MF}}}{w}_{ij}^{\mathrm{MF}\to \mathrm{PC}}{\mathrm{MF}}_i^{p,d}(t) $$

Here we used a notation that hatted firing rates were estimates whereas nonhatted variables were experimentally observed ones. ${\left({\mathbf{w}}^{\mathrm{MF}\to \mathrm{PC}}\right)}_{ij}={w}_{ij}^{\mathrm{MF}\to \mathrm{PC}}$ is the *N*_PC_ × *N*_MF_ weight matrix, that is, for each PC, there are *N*_MF_ adjustable weights. Note that these weights cannot be interpreted directly as synaptic strengths between MFs and PCs because they are indirectly connected via granule cells as well as inhibitory interneurons. The weights should rather be interpreted not as anatomical but functional connections between MFs and PCs. The weights are assumed to be either positive or negative, reflecting the anatomical fact that, between MFs and PCs, there are excitatory (granule cells) and inhibitory interactions (Fig. 1 inset). The weight matrix was optimized so as to minimize a squared error between actual firing rate PC_*i*_(*t*) and reconstructed firing rates ${\widehat{\mathrm{PC}}}_i(t)$ averaged over experimental duration and movement directions as

4 $$ \frac{1}{2}\sum \limits_d\sum \limits_{-1\le t\le 1}{\left\{{\mathrm{PC}}_i^{p,d}(t)-{\widehat{\mathrm{PC}}}_i^{p,d}(t)\right\}}^2 $$

The optimization was performed for the two postures separately so that we investigated how the weights trained for one posture generalized to the other posture. Note that the reconstructed firing rates of a PC in one condition (i.e., movement direction and posture) used those of MFs in the same condition. For optimizing the weights, the entire experimental duration [− 1 s, + 1 s] was used. The fitting to the data was evaluated by computing the coefficient of determination (*R*^2^) in a time window of [− 0.2 s, + 0.5 s] because task-related modulation of firing rates occurred mainly in this time window.

### Linear Reconstruction of Firing Rates of DCs

It is known from the cerebellar anatomy that DCs receive excitatory inputs from MFs and inhibitory inputs from PCs, respectively. As in the case for PCs, firing rates of a DC were reconstructed as a weighted sum of those of MFs and PCs,

5 $$ {\widehat{\mathrm{DC}}}_i^{p,d}(t)=\sum \limits_{j=1}^{N_{\mathrm{MF}}}{w}_{ij}^{\mathrm{MF}\to \mathrm{DC}}{\mathrm{MF}}_j^{p,d}(t)+\sum \limits_{k=1}^{N_{\mathrm{PC}}}{w}_{ik}^{\mathrm{PC}\to \mathrm{DC}}{\mathrm{PC}}_k^{p,d}(t) $$

Collaterals of MFs send excitatory inputs and PCs send inhibitory inputs to DCs, so $\left\{{w}_{ij}^{\mathrm{MF}\to \mathrm{DC}}\right\}$ and $\left\{{w}_{ik}^{\mathrm{PC}\to \mathrm{DC}}\right\}$ were assumed to be nonnegative and nonpositive, respectively. For each DC, the linear model contained *N*_MF_ + *N*_PC_ weights, and the squared error between DC_*i*_(*t*) and ${\widehat{\mathrm{DC}}}_i(t)$ was minimized under the nonnegative and nonpositive constraints. These weights were optimized using a standard quadratic programming algorithm.

Additionally, to investigate how the MF and PC firings contributed to the reconstruction of DC firings, we attempted to reconstruct the DC firings using only the MF firings as in

6 $$ {\widehat{\mathrm{DC}}}_i^{p,d}(t)={w}_i^{\mathrm{MF}\to \mathrm{DC}}+\sum \limits_{j=1}^{N_{\mathrm{MF}}}{w}_{ij}^{\mathrm{MF}\to \mathrm{DC}}{\mathrm{MF}}_j^{p,d}(t) $$

or using only the PC firings as in.

7 $$ {\widehat{\mathrm{DC}}}_i^{p,d}(t)={w}_i^{\mathrm{PC}\to \mathrm{DC}}+\sum \limits_{k=1}^{N_{\mathrm{PC}}}{w}_{ik}^{\mathrm{PC}\to \mathrm{DC}}{\mathrm{PC}}_k^{p,d}(t) $$

To explain spontaneous activities of DCs, the constant terms (${w}_i^{\mathrm{MF}\to \mathrm{DC}}$ in Eq. (6) and ${w}_i^{\mathrm{PC}\to \mathrm{DC}}$ in Eq. (7)) were included. Values of the goodness-of-fit using both MF and PC firings (Eq. (5)), MF firings only (Eq. (6)), or PC firings only (Eq. (7)) were compared.

### Statistical Tests of Linear Reconstruction

To test statistical significance of linear reconstructions of PCs, two tests were performed. First, we assessed the goodness-of-fit of the linear model to the data by comparing other models. This was a model comparison *within* a posture. Two standard nonlinear models (a threshold model and a quadratic model) were fit to the data. One was a linear-threshold model where the MF firing rates are thresholded by zeroing the activity equal to or smaller than a threshold as

8 $$ {\widehat{\mathrm{PC}}}_i(t)=\sum \limits_{j=1}^{N_{\mathrm{MF}}}{w}_{ij}^{\mathrm{MF}\to \mathrm{PC}}{\left[{\mathrm{MF}}_j(t)-{\theta}_j\right]}_{+} $$

Here *θ*_*j*_ denotes an activity threshold of *j*-th MF. The linear-threshold model is often used for modeling nonlinear amplification, multiplicative gain modulation, and winner-takes-all selection [16]. In our context, the thresholding operation may be regarded as a nonlinear processing of granule cells between MFs and PCs. The other was a quadratic model which is analogous to an energy model of complex cells in the visual cortex [17]:

9 $$ {\widehat{\mathrm{PC}}}_i(t)=\sum \limits_{j=1}^{N_{\mathrm{MF}}}{w}_{ij}^{\mathrm{MF}\to \mathrm{PC}}{\mathrm{MF}}_j(t)+\sum \limits_{j=1}^{N_{\mathrm{MF}}}{v}_{ij}^{\mathrm{MF}\to \mathrm{PC}}{\left[{\mathrm{MF}}_j(t)-{\theta}_j\right]}_{+}^2 $$

This model has been used for modeling the properties of complex cells whose responses are orientation selective but not phase selective. The above models are instantaneous, i.e., the firing rate of a PC at time *t* is reconstructed with that of a MF at the same time *t*. In addition, we also considered a finite-impulse-response (FIR) model of order 1 defined as

10 $$ {\widehat{\mathrm{PC}}}_i^{p,d}(t)=\sum \limits_{j=1}^{N_{\mathrm{MF}}}{w}_{ij}^{(0)\ \mathrm{MF}\to \mathrm{PC}}{\mathrm{MF}}_i^{p,d}(t)+\sum \limits_{j=1}^{N_{\mathrm{MF}}}{w}_{ij}^{(1)\ \mathrm{MF}\to \mathrm{PC}}{\mathrm{MF}}_i^{p,d}\left(t-1\right) $$

The second term may be interpreted as a reverberation in the recurrent circuit composed of MFs, granule cells, and Golgi cells, so this FIR model is the simplest example of the adaptive filter model.

For a single PC cell, the linear, the threshold, and the quadratic models contain *N*_MF_, 2*N*_MF_, and 3*N*_MF_ adjustable parameters, respectively. For each cell, the three models were compared according to the AIC. In the same way, the linear reconstruction model of DCs was tested with a linear-threshold model,

11 $$ {\widehat{\mathrm{DC}}}_i^{p,d}(t)=\sum \limits_{j=1}^{N_{\mathrm{MF}}}{w}_{ij}^{\mathrm{MF}\to \mathrm{DC}}\left[{\mathrm{MF}}_j^{p,d}(t)-{\theta}_j\right]+\sum \limits_{k=1}^{N_{\mathrm{PC}}}{w}_{ik}^{\mathrm{PC}\to \mathrm{DC}}\left[{\mathrm{PC}}_k^{p,d}(t)-{\varphi}_k\right] $$

and a quadratic model,

12 $$ {\displaystyle \begin{array}{l}{\widehat{\mathrm{DC}}}_i^{p,d}(t)=\sum \limits_{j=1}^{N_{\mathrm{MF}}}{w}_{ij}^{\mathrm{MF}\to \mathrm{DC}}{\mathrm{MF}}_j^{p,d}(t)+\sum \limits_{j=1}^{N_{\mathrm{MF}}}{w}_{ij}^{-\mathrm{MF}\to \mathrm{DC}}{\left[{\mathrm{MF}}_j^{p,d}(t)-{\theta}_j\right]}^2\\ {}\kern4.25em +\sum \limits_{k=1}^{N_{\mathrm{PC}}}{w}_{ik}^{\mathrm{PC}\to \mathrm{DC}}{\mathrm{PC}}_k^{p,d}(t)+\sum \limits_{k=1}^{N_{\mathrm{PC}}}{w}_{ik}^{-\mathrm{PC}\to \mathrm{DC}}{\left[{\mathrm{PC}}_k^{p,d}(t)-{\varphi}_k\right]}^2\end{array}} $$

The linear, linear-threshold, and quadratic models contain *N*_MF_ + *N*_PC_, 2*N*_MF_ + 2*N*_PC_, and 3*N*_MF_ + 3*N*_PC_ parameters, respectively. As in Eq. (9), $\left\{{w}_{ij}^{\mathrm{MF}\to \mathrm{DC}}\right\}$ and $\left\{{w}_{ik}^{\mathrm{PC}\to \mathrm{DC}}\right\}$ were constrained to be nonnegative and nonpositive, respectively, and other parameters were unconstrained. These models were also compared according to the AICs.

Second, how the reconstruction of linear and other models learned at one posture generalized to those at the other posture. Here, we expected that a reconstruction model that appropriately describes the firing rates should reconstruct not only at a trained posture but also at an untrained posture. Hence, this was a model comparison *across* the two postures. The degree of generalization was evaluated for the four models of PC firing-rate fitting and for the three models of DC firing-rate fitting. Specifically, for one target cell, the weights optimized at one posture were used for a linear reconstruction at the other posture, and the goodness-of-fit between the linear reconstruction and the data was computed at the untrained posture. The goodness-of-fit of multiple models was statistically compared by one-way ANOVA.

### Statistical Comparison of MF–PC and MF–DC Projections

MFs project both to PCs via granule cells and parallel fibers and to DCs. Correspondingly, the linear equations we consider include the two terms $\left\{{w}_{ij}^{\mathrm{MF}\to \mathrm{PC}}\right\}$ in Eq. (3) and $\left\{{w}_{ij}^{\mathrm{MF}\to \mathrm{DC}}\right\}$ in Eq. (5), which represent functional projections from MFs and PCs and from MFs to DCs, respectively. Each projection was characterized by a column of $\left\{{w}_{ij}^{\mathrm{MF}\to \mathrm{PC}}\right\}$ or $\left\{{w}_{ij}^{\mathrm{MF}\to \mathrm{DC}}\right\}$, which we refer to a projection vector. Similarity of projections was quantified by computing a correlation coefficient between two projection vectors. Specifically, we asked whether the two functional projections statistically differed. Correlation coefficients of all possible pairs of MF–PC projection vectors and MF–DC projection vectors were computed, and their average served as an index of similarity between the two projections. Note that the MF–PC projection vectors took either positive or negative values while the MF–DC projection vectors were nonnegative. Absolute values of the MF–PC projection vectors were used for the computation of correlation coefficients because only the magnitudes of projections were of our interest. To assess the statistical significance of the average of correlation coefficients, a resampling test was performed based on a null hypothesis that there was no statistical difference between MF–PC and MF–DC projections. Specifically, a bootstrap distribution of correlation coefficients was computed by randomly permuting the labels of projection vectors for 100,000 times. The probability of observing the average of correlation coefficients was assessed in terms of the bootstrap distribution.

### Linear Predictions of Future MF Activities from Current DC Activities

To test the forward-model hypothesis, we then explored to investigate whether the current outputs from the cerebellum (i.e., DCs) could predict the future inputs to the cerebellum (i.e., MFs). Specifically, the firing rates of each MF at time *t* + *t*_1_ were reconstructed as a weighted sum of the firing rates of DCs at time *t*, as

13 $$ {\widehat{\mathrm{MF}}}_i\left(t+{t}_1\right)=\sum \limits_{j=1}^{N_{\mathrm{DC}}}{w}_{ij}^{\mathrm{DC}\to \mathrm{MF}}{\mathrm{DC}}_j(t) $$

This linear prediction model contained *N*_DC_ weights for one MF. As in the linear reconstruction cases, the squared error between the predicted and the actual firing rates was minimized to compute the optimal values of the weights. The weights $\left\{{w}_{ij}^{\mathrm{DC}\to \mathrm{MF}}\right\}$ cannot be interpreted as functional connectivity from DCs to MFs, as there are no anatomically direct connections between DCs and MFs. Rather, the linear prediction model was introduced to test if the current DC activity contained predictive information about the future MF activity. *t*_1_ is a parameter of time advance and was varied from 0 to 200 ms in steps of 20 ms (the window of time bins). The goodness-of-fit of the linear model was evaluated in a time window of [− 200 ms, 500 ms].

One may suspect that the linear prediction in Eq. (13) is possible if the terms of the right-hand side span a wide variety of waveforms, as in the case of Fourier expansion whereby any time series can be fit with a set of sinusoidal waveforms. To assess the fit statistically and to exclude that possibility, a bootstrap test was performed by shuffling the movement directions of DCs on the right-hand side of Eq. (13), while the movement directions of MFs on the left-hand side were fixed to those used experimentally. Randomizing the movement directions on the right-hand side maintained the diversity of waveforms but eliminated directional relationship between both sides of Eq. (13). The bootstrap test was designed based on a null hypothesis that any time series of the same degree of similarity could predict the future inputs to the cerebellum. With one sequence of shuffled targets, the goodness-of-fit was computed for all MFs and then averaged. This was repeated for 10,000 times to produce a bootstrap distribution of goodness-of-fit, and then the probability of the experimental goodness-of-fit was computed. This bootstrap test was performed with *t*_1_ ranging from 20 to 100 ms in step of 20 ms for the two postures, separately.

### Sparse Linear Analyses

Finally, to further investigate what connectivity structure underlies the linear reconstruction and prediction described above, a sparse linear analysis was performed. Specifically, we aimed to discover how many nonzero weights sufficed to reconstruct or predict neural firing patterns of a target cell. In addition to the squared errors between actual and reconstructed firing rates, sparsity imposing terms were incorporated. Specifically, an L1 norm of a weight vector (i.e., a sum of absolute values of weight coefficients) was used by minimizing the following cost functions,

14 $$ \frac{1}{2T}\sum \limits_{-1\le t\le 1}{\left\{{\mathrm{PC}}_i(t)-\sum \limits_{j=1}^{N_{\mathrm{MF}}}{w}_{ij}^{\mathrm{MF}\to \mathrm{PC}}{\mathrm{MF}}_j(t)\right\}}^2+\lambda \sum \limits_{j=1}^{N_{\mathrm{MF}}}\mid {w}_{ij}^{\mathrm{MF}\to \mathrm{PC}}\mid $$

15 $$ \frac{1}{2T}\sum \limits_{-1\le t\le 1}{\left\{{\mathrm{DC}}_i(t)-\sum \limits_{j=1}^{N_{\mathrm{MF}}}{w}_{ij}^{\mathrm{MF}\to \mathrm{DC}}{\mathrm{MF}}_j(t)-\sum \limits_{k=1}^{N_{\mathrm{DC}}}{w}_{ik}^{\mathrm{PC}\to \mathrm{DC}}{\mathrm{PC}}_k(t)\right\}}^2+\lambda \left(\sum \limits_{j=1}^{N_{\mathrm{MF}}}{w}_{ij}^{\mathrm{MF}\to \mathrm{DC}}-\sum \limits_{j=1}^{N_{\mathrm{MF}}}{w}_{ij}^{\mathrm{MF}\to \mathrm{PC}}\right) $$

16 $$ \frac{1}{2T}\sum \limits_{-1\le t\le 1}{\left\{{\mathrm{MF}}_i\left(t+{t}_1\right)-\sum \limits_{j=1}^{N_{\mathrm{DC}}}{w}_{ij}^{\mathrm{DC}\to \mathrm{MF}}{\mathrm{DC}}_j(t)\right\}}^2+\lambda \sum \limits_{j=1}^{N_{\mathrm{DC}}}\mid {w}_{ij}^{\mathrm{DC}\to \mathrm{MF}}\mid $$

Here *T* denotes the length of data. Note that Eqs. (14) and (16) are the cost functions used in a standard sparse linear analysis known as LASSO [18]. Equation (15) slightly differs from LASSO because of the nonnegative constraint of MF → DC weights and the nonpositive constraint of PC → DC weights. The time advance parameter *t*_1_ in Eq. (16) was fixed to 40 ms in this sparse analysis. The parameter *λ* determines the tradeoff between the squared error and the sparseness and must be optimized for individual target cells. For each target cell, we varied *λ* in a range from 0.05 to 5 and chose the value that exhibited the smallest generalization error for test data in tenfold cross validation. The weight coefficients computed with the optimized *λ* were assessed in two ways: the proportion of nonzero weights and the proportion of significantly contributing weights. Here, the significantly contributing weights were defined by counting the number of weights whose cumulative sum exceeded 90% of the sum of total weights.

## Results

First, statistical characteristics of firing rates of the three populations were computed and compared (see “Characterization of Firing Rates: Spatiotemporal Separability Index and Distributions” in the “Methods” section). Then, the firing rates of PCs and DCs were reconstructed with linear weighted models of MF and PC firing rates (see “Linear Reconstruction of Firing Rates of PCs” and “Linear Reconstruction of Firing Rates of DCs” in the “Methods” section). The reconstructions of the linear models were statistically compared to those of nonlinear and FIR models by computing AICs within a single posture and the degree of generalization across two postures (see “Statistical Test of Linear Reconstruction”). Finally, the forward-model hypothesis was tested by applying a linear-weighted model that predicted the MF activity at time *t* + *t*_1_ from the DC activity at time *t* (see “Linear Prediction of Future MF Activities from Current DC Activities” in the “Methods” section).

### Characterization of Firing Rates

The STSI, defined in Eq. (2), was introduced to assess the complexity of spatiotemporal patterns of firing rates of a given neuron (see “Characterization of Firing Rates: Spatiotemporal Separability Index and Distributions” in the “Methods” section). STSI exhibited clearly separable values for MFs and PCs (Fig. 1a). Our previous papers reported that task-related activities recorded from MFs showed a unimodal and directionally tuned phasic modulation around movement onset that was analogous to those observed in activities of the primary motor cortex (M1) [19–21], and that simple-spike activities of PCs showed dynamic and time-varying directional tuning before and after movement onset [14, 15]. Consistent with these observations, STSIs of MFs were significantly larger than STSIs of PCs (unpaired *t* test, *p* < 0.01). The population of MFs had the largest value (median 0.63 ± 0.14 SD), followed by the population of DCs (median 0.48 ± 0.15 SD), and the population of PCs had the smallest values (median 0.38 ± 0.15 SD). There was a statistically significant difference between STSIs of the three populations as determined by one-way ANOVA (*F*(2, 497) = 95.3, *p* = 9.18 × 10^−36^). A Tukey post hoc test revealed that the STSIs of MFs were statistically significantly larger than those of PCs (*p* = 9.6 × 10^−10^) and those of DCs (*p* = 9.6 × 10^−10^) and that the STSIs of DCs were statistically larger than those of PCs (*p* = 2.7 × 10^−5^). Therefore, the firing rates of MFs exhibited spatiotemporally separable and simple characteristics, the firing rates of PCs exhibited spatiotemporally nonseparable and complex characteristics, and the firing rates of DCs exhibited characteristics intermediate between MFs and PCs.

The distributions of firing rates were computed for the three populations, respectively (Fig. 1b). They were unimodal (MF: mean 21.1 ± 17.6 SD; PC: mean 48.2 ± 22.7; DC: mean 38.6 ± 22.7). These distributions were fitted with Gaussian, Gamma, Rayleigh, and inverse Gaussian distributions, which were compared according to the values of AICs. We found that the Gamma distribution provided the smallest values of AICs for the MF (1.26 × 10^6^ (Gaussian), 1.18 × 10^6^ (Gamma), 1.25 × 10^6^ (Rayleigh), 5.5 × 10^6^ (inverse Gaussian)), PC (1.201 × 10^6^ (Gaussian), 1.191 × 10^6^ (Gamma), 1.194 × 10^6^ (Rayleigh), 1.216 × 10^6^ (inverse Gaussian)), and DC firing rates (1.045 × 10^6^ (Gaussian), 1.023 × 10^6^ (Gamma), 1.025 × 10^6^ (Rayleigh), 1.052 × 10^6^ (inverse Gaussian)). A Gamma distribution has a property that a sum of two independent random variables from a Gamma distribution obeys also a Gamma distribution. Actual neural activities are correlated to each other, so the fact that the firing rates were all distributed as Gamma distributions does not necessarily support simple summation from one population to another, but rather imply some simple transformations among the populations.

The two analyses above found that the three populations had distinct degrees of spatiotemporal complexity as quantified in terms of STSIs, while the firing rates were all Gamma distributed. These results, taken together, led us to a hypothesis that the transformation from one population to another might be linear, so we went on to test the hypothesis by linearly fitting the firing rates of one population with those of preceding populations.

### Linear Reconstruction of Firing Rates of PCs and DCs

**Fig. 2:**
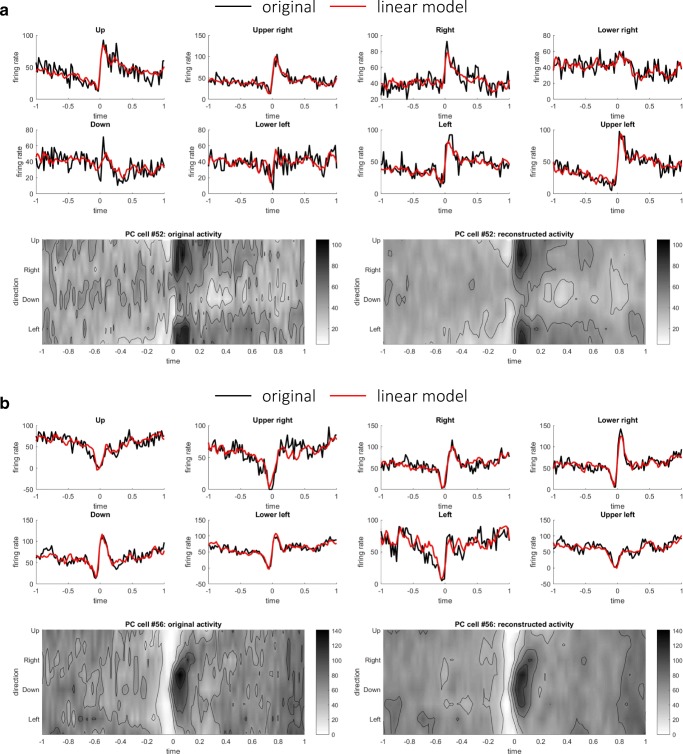
Two representative examples of linear reconstructions of PCs. **a** Original and reconstructed firing rates of a representative PC (#52). Eight upper panels compare time series of the original (black) and the reconstructed (red) firing rates for each movement direction. *R*^2^ is 0.961 for this PC. Two lower panels provide the same firing rates of the original (left) and the reconstructed (right) firing rates in contour plots. **b** Original and reconstructed firing rates of another representative PC (#56), presented in the same format of **a**. *R*^2^ is 0.964 for this PC

Based on the results of STSI and distributions of firing rates, we inferred that the firing rates of PCs and DCs might consist of weighted sum of the firing rates of input populations. We here attempted to reconstruct the firing rates of PCs as weighted sums of those of MFs using a linear model (3) (see “Linear Reconstruction of Firing Rates of PCs” in the “Methods” section). The weight parameters were optimized so that the squared error between actual firing rate PC_*i*_(*t*) and reconstructed firing rates ${\widehat{\mathrm{PC}}}_i(t)$ (Eq. (4)) was minimized separately for the pronated and supinated postures. The results of the two postures were similar; we present the results of the pronated posture below. Figure 2 illustrates time series and contour plots of the firing rates of two representative PCs that exhibited the highest *R*^2^ values between the original and reconstructed firing rates. In one PC (Fig. 2a), firing rates underwent a suppression just before the movement onset and an increase after the movement onset around movement directions 1 and 8, therefore exhibiting the reversal of its preferred direction. The reconstructed firing rates captured the reversal of preferred direction. In another PC (Fig. 2b), there was a uniform suppression of firing rates over the movement direction before the movement onset, followed by the emergence of directional tuning around directions 4 and 5 after the movement onset. As a population, the linear reconstruction model explained the original firing rates of PCs, as evidenced by *R*^2^ values for the pronated posture (mean 0.95 ± 0.023 SD) and the supinated posture (mean 0.96 ± 0.023 SD).

**Fig. 3:**
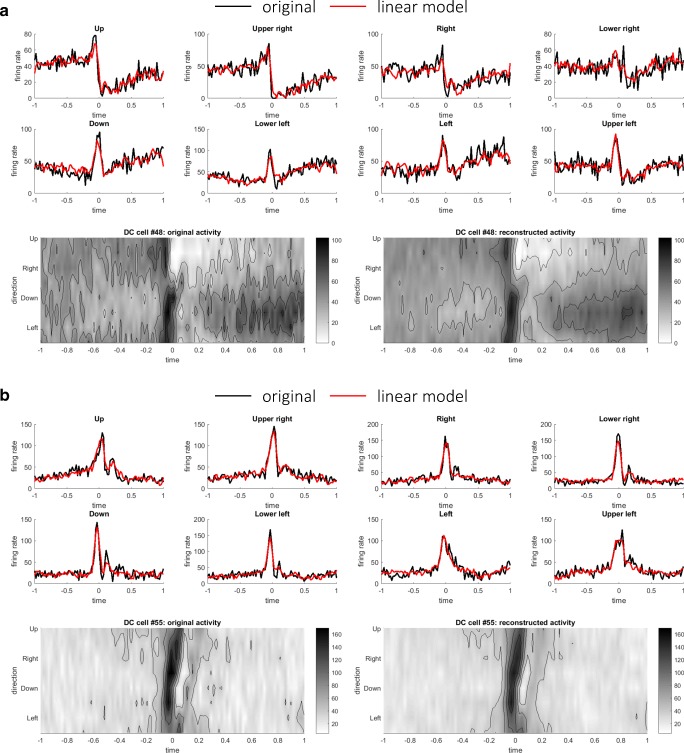
Two representative examples of linear reconstructions of DCs: **a** DC cell #48 (*R*^2^ value 0.95) and **b** DC cell #55 (*R*^2^ value 0.96), in the same format of Fig. 2

To further examine the hypothesis, we then proceeded to reconstruct the firing rates of DCs as linear weighted sums of MFs and PCs using Eq. (5) (see “Linear Reconstruction of Firing Rates of DCs” in the “Methods” section). The firing rates of DCs were reconstructed as weighted sum of excitatory (nonnegative) inputs from MFs and inhibitory (nonpositive) inputs from PCs (Eq. (5)). Figure 3 depicts two representative DCs that exhibited the highest *R*^2^ values between the original and reconstructed firing rates. As in the PC case, the firing rates of DCs were well reconstructed as linear sums of MFs and DCs. As a population, the median values of *R*^2^ of linear model were 0.94 ± 0.048 SD for the pronated posture and 0.93 ± 0.0420 SD for the supinated posture. For a comparison, the DC firing rates were reconstructed using only the MF or PC firings minimizing Eq. (6) or (7), respectively. The median values of *R*^2^ using only MFs were 0.92 ± 0.069 (pronated) and 0.91 ± 0.067 (supinated), and the median values of *R*^2^ using only MFs were 0.91 ± 0.060 (pronated) and 0.91 ± 0.050 (supinated). There was a statistically significant difference between the *R*^2^ values of the linear reconstructions using MF + PC, MF only, or PC only as determined by one-way ANOVA (*F*(2, 216) = 5.02, *p* = 7.4 × 10^−3^ (pronated); *F*(2, 216) = 6.82, *p* = 1.3 × 10^−3^ (pronated)). A Tukey post hoc test revealed that the *R*^2^ values of MF + PC reconstructions were significantly better than those of MF reconstructions or those of PC reconstructions (*p* = 0.047 for MF only and *p* = 0.008 for PC only (pronated), *p* = 0.0098 for MF only and *p* = 0.0018 for PC only (supinated)).

### Comparison of AIC for Multiple Models of PCs and DCs

**Fig. 4:**
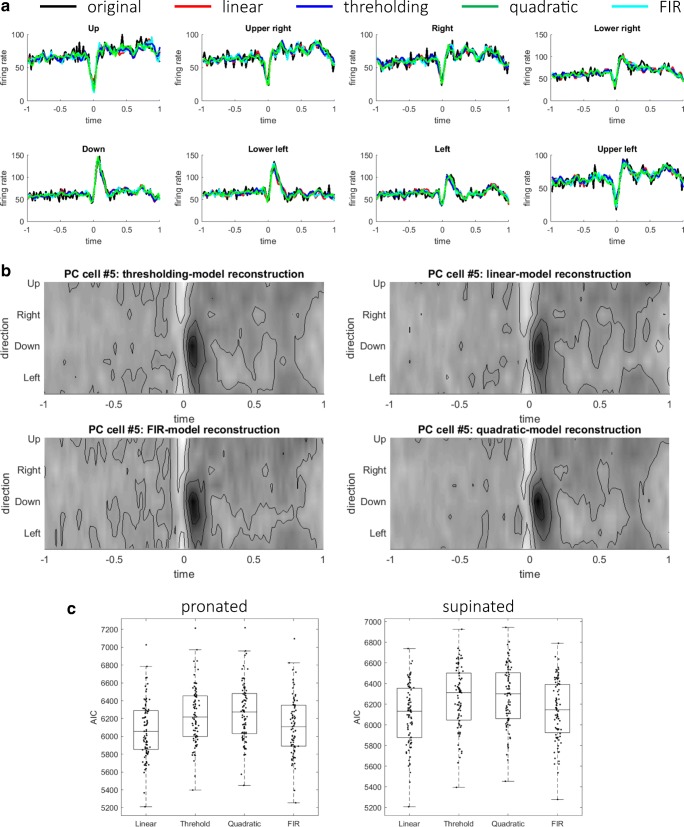
Comparison of Akaike information criterion (AIC) between the linear, threshold, quadratic, and FIR models for PC cell fitting. A typical example of model fitting to the firing rates of a PC (#52) shown in **a** time series and **b** contour plots. In **a**, the firing rates of the original, linear, threshold, quadratic, and FIR models are shown with black, red, blue, green, and cyan lines, respectively. **c** Box plots of the goodness-of-fit for the four models at the pronated (left) and supinated (right) postures. On each box, the central mark is the median, and the edges of the box are the 25th and 75th percentiles, respectively

The quality of linear reconstruction indicated that the firing rates of MFs and PCs contributed linearly and instantaneously to those of PCs and DCs. Previous models of the cerebellum, on the contrary, suggest nonlinear or noninstantaneous models; the perceptron model assumes thresholding nonlinearity, and the adaptive filter model assumes finite impulse responses that depend on not only current inputs but also previous inputs. To verify whether the linear reconstruction sufficed or not, two simple nonlinear reconstruction models (a threshold model (8) and a quadratic model (9)) and an FIR model of order 1 (10) were fitted to the firing rates of PCs (see “Statistical Test of Linear Reconstruction”). The results of the two postures were similar; the results of the pronated posture are shown here. A typical PC reconstruction was presented in the format of time series (Fig. 4a) and contour plots (Fig. 4b). A visual inspection suggested that the reconstruction of the threshold model was indistinguishable from that of the linear model and that the reconstructions of quadratic model and FIR model were marginally better. The values of AIC are summarized in Fig. 4c and Table 1. As a population, AIC took the smallest value for the linear model and the second smallest value for the FIR model. For individual PCs, the values of AIC were minimum for the linear model for a majority of PCs (77 for pro and 71 for sup) and for the FIR model for a minority of PCs (6 for pro and 12 for sup). The thresholding model or the quadratic model was not selected for any PCs. Therefore, the linear model best captured the relationship between firing rates of MFs and PCs.

**Table 1:** Summary of AIC statistics for PC firing-rate fitting

	Linear model	Thresholding model	Quadratic model	FIR model
Median (STD) for pro posture	6.05 × 10^3^(3.19 × 10^2^)	6.22 × 10^3^(3.23 × 10^2^)	6.27 × 10^3^(3.23 × 10^2^)	6.11 × 10^3^(3.23 × 10^2^)
Median (STD) for sup posture	6.10 × 10^3^(2.99 × 10^2^)	6.26 × 10^3^(3.02 × 10^2^)	6.27 × 10^3^(2.97 × 10^2^)	6.14 × 10^3^(2.97 × 10^2^)

**Fig. 5:**
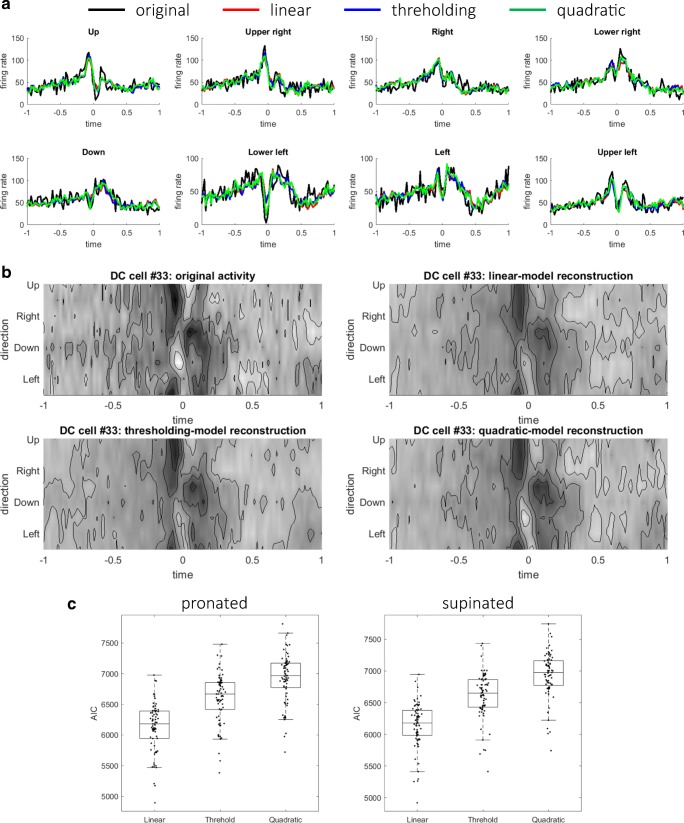
Comparison of AIC between the linear, threshold, and quadratic models for DC cell fitting. A typical example of model fitting to the firing rates of a DC (#33) shown in time series (**a**) and contour plots (**b**). In **a**, the firing rates of the original, linear, threshold, and quadratic models are shown with black, red, blue, and green lines, respectively. **c** Box plots of the goodness-of-fit for the three models at the pronated (left) and supinated (right) postures

Similarly, for DC firing-rate fitting, the linear model fitting was statistically compared with those of two nonlinear models (the thresholding model (11) and the quadratic model (12)). The linear and nonlinear models were fitted to the firing rates of DCs, and AIC was computed for all DCs. A typical DC reconstruction was presented in the format of time series (Fig. 5a) and contour plots (Fig. 5b). The three models reconstructed the original activities at almost the same quality. The values of AIC are summarized in Fig. 5c and Table 2. The comparison of AIC indicated that the linear model could explain the DC firings most parsimoniously for all the DCs. Again, the linear model best described the firing rates of DCs with firing rates of MFs and PCs. In summary, the linear reconstruction model was selected for both reconstructions of PC and DC firing rates.

**Table 2:** Summary of AIC statistics for DC firing-rate fitting

	Linear model	Thresholding model	Quadratic model
Median (STD) for pro posture	6.18 × 10^3^ (4.01 × 10^2^)	6.67 × 10^3^ (4.01 × 10^2^)	6.97 × 10^3^ (3.98 × 10^2^)
Median (STD) for sup posture	6.18 × 10^3^ (3.62 × 10^2^)	6.65 × 10^3^ (3.63 × 10^2^)	6.97 × 10^3^ (3.59 × 10^2^)

### Generalization Across Postures of Fitted Models of PCs and DCs

**Fig. 6:**
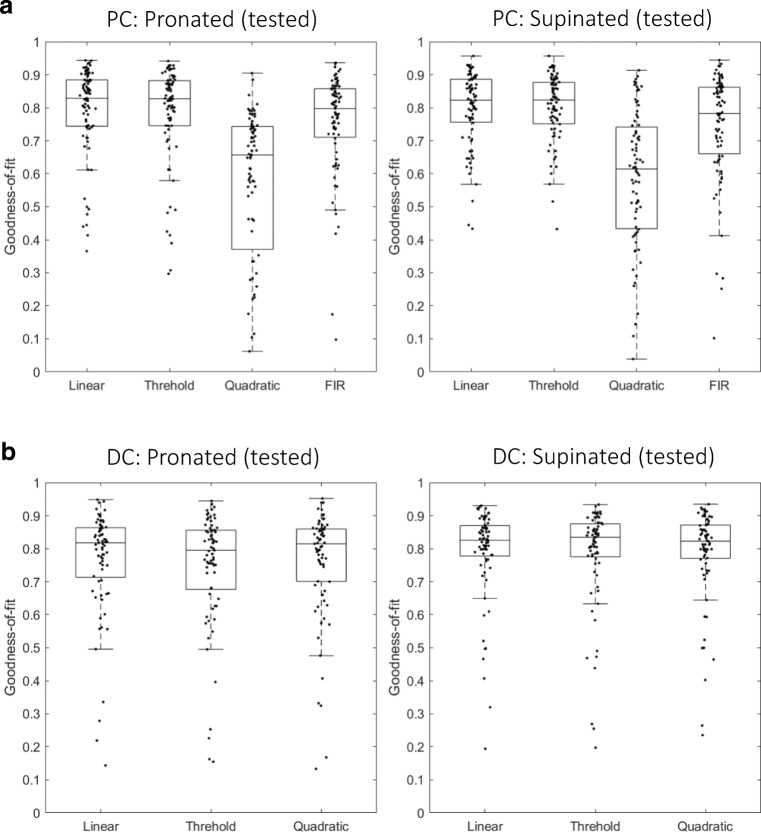
Generalization from fitting at one posture to another posture of multiple models. **a** Box plots of goodness-of-fit (left) trained in supinated posture and tested in pronated posture and (right) trained in pronated posture and tested in supinated posture for PCs. **b** Box plots of goodness-of-fit (left) trained in supinated posture and tested in pronated posture and (right) trained in pronated posture and tested in supinated posture for DCs

The AIC test was performed to select the most parsimonious model *within* a posture. We then proceeded to ask which model best generalized firing-rate fitting *across* two postures (see “Statistical Test of Linear Reconstruction”). Specifically, the weights trained in one posture were used to reconstruct the firing rates in the other posture that were not used for training. The degrees of generalization were summarized in box plots of Fig. 6a: (left panel) from supinated to pronated posture, and (right panel) from pronated to supinated posture, and Table 3. The goodness-of-fit was almost equal for the linear, threshold, and FIR models. In contrast, the goodness-of-fit was significantly worse for the quadratic model than those for the other models, indicating that the quadratic model overfit to the trained posture and did not generalize properly to the untrained posture. There was a statistically significant difference between the four models as determined by one-way ANOVA (*F*(3, 328) = 19.8, *p* = 7.57 × 10^−12^ (trained in pro and tested in sup); *F*(3, 328) = 20.9, *p* = 2.1 × 10^−12^ (trained in sup and tested in pro)). A Tukey post hoc test revealed that the goodness-of-fit of the quadratic model was significantly worse than the three models (*p* = 3.6 × 10^−9^ for the linear model, *p* = 5.1 × 10^−9^ for the thresholding model, and *p* = 1.3 × 10^−7^ for the FIR model (trained in pro and tested in sup); *p* = 3.8 × 10^−9^ for the linear model, *p* = 3.8 × 10^−9^ for the thresholding model, and *p* = 1.4 × 10^−7^ for the FIR model (trained in sup and tested in pro)). There was no significant difference between the linear, thresholding, and FIR models. In summary, the linear, thresholding, and FIR models generalized from one posture to another almost equally, whereas the generalization of the quadratic model was significantly worse than those of the other models.

**Table 3:** Summary of degrees of generalization across two postures for PC firing-rate fitting

	Linear model	Thresholding model	Quadratic model	FIR model
Fig. 6a, PC: pronated (trained in supinated) Median (SD)	0.82 (0.18)	0.82 (0.17)	0.61 (0.34)	0.78 (0.17)
Figure 6a, PC: supinated (trained in pronated) Median (SD)	0.83 (0.19)	0.83 (0.12)	0.66 (0.37)	0.78 (0.20)

Next, we investigated how the weights trained in one posture reconstructed the DC activities in another posture, as summarized in Fig. 6b (left panel) from supinated to pronated posture, and (right panel) from pronated to supinated posture and Table 4. The goodness-of-fit was almost equal for the three models. There was no statistically significant difference between the three models as determined by one-way ANOVA (*F*(2, 246) = 0.10, *p* = 0.90 (trained in pro and tested in sup); *F*(2,246) = 2.5 × 10^−3^, *p* = 0.9975 (trained in sup and tested in pro)). Therefore, the degree of generalization from one posture to another was not different between the three models.

**Table 4:** Summary of degrees of generalization across two postures for PC firing-rate fitting

	Linear model	Thresholding model	Quadratic model
Fig. 6b, DC: pronated (trained in supinated) Median (SD)	0.81 (0.29)	0.82 (0.29)	0.81 (0.29)
Fig. 6b, DC: supinated (trained in pronated) Median (SD)	0.80 (0.29)	0.78 (0.29)	0.80 (0.29)

### Statistical Comparison of MF–PC and MF–DC Projections

In the linear equations derived from the experimental firing rates, there are two distinct projections from the MFs: from MFs to PCs (*w*^MF → PC^ in (3)) and from MFs to DCs (*w*^MF → DC^ in (5)) (see “Statistical Comparison of MF–PC and MF–DC Projections” in the “Methods” section). We asked whether a common population of MFs projected both to PCs and DCs or separate populations of MFs projected to PCs and DCs. The average of correlation coefficients between MF–PC and MF–DC projection vectors was 0.060. We assessed a statistical significance of this value of correlation by a resampling test with a null hypothesis that there was no statistical difference between the two types of projections. A 99% confidence interval was [0.1027, 0.1089], and the average value of experimental correlation coefficients was significantly small (*p* < 10^−5^). Therefore, the null hypothesis was rejected, and PCs and DCs did not receive projections from the same population of MFs.

### Linear Predictions of Future Inputs from Current Outputs

**Fig. 7:**
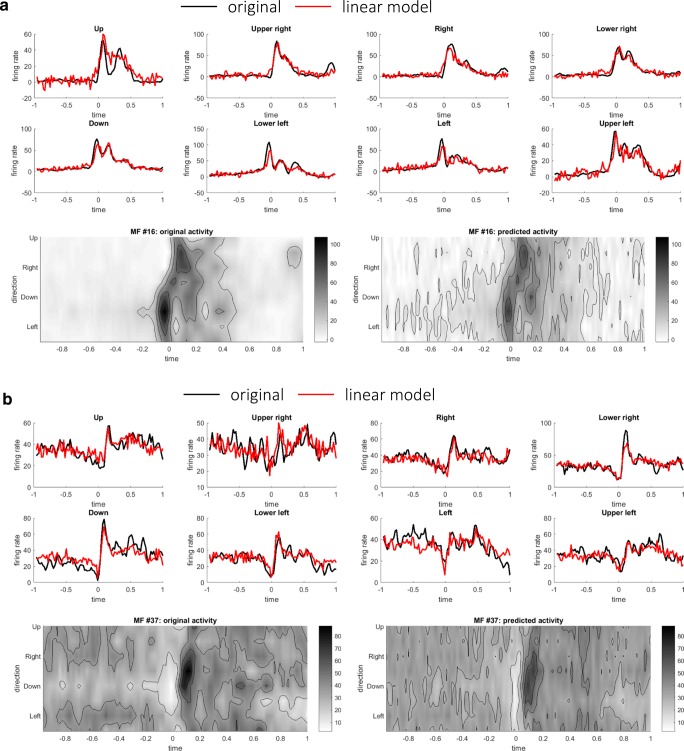
Two representative examples of linear predictions of MFs (#16 in **a** (*R*^2^ value 0.91) and #37 in **b** (*R*^2^ value 0.93)) at time *t* + *t*_1_ from DCs at time *t*. Here *t*_1_ was set to 40 ms. In each panel, the original activities and the predicted activities were compared in terms of time series and contour plots

**Fig. 8:**
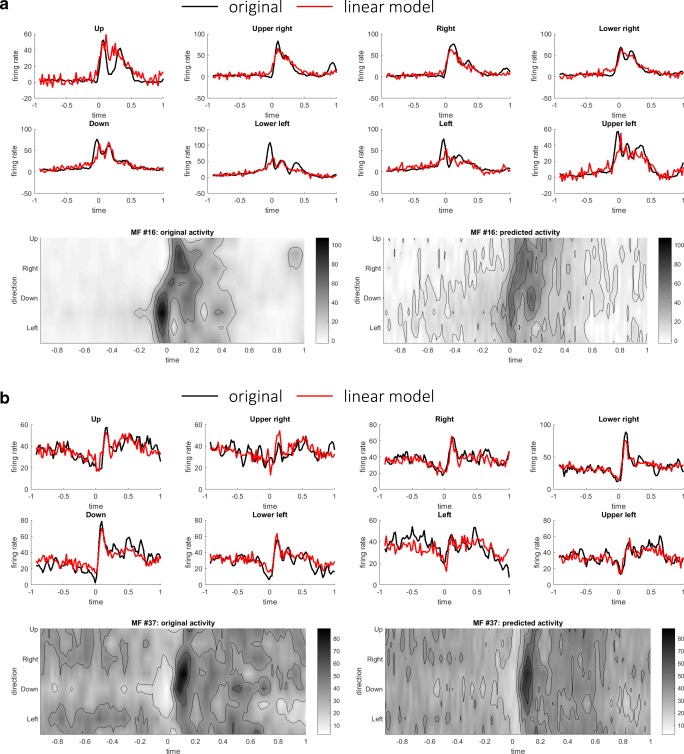
Two representative examples of linear predictions of MFs at time *t* + *t*_1_ from DCs at time *t*. These are the same MFs (#16 in **a** (*R*^2^ value 0.85) and #37 in **b** (*R*^2^ value 0.89)) presented in Fig. 7. Here *t*_1_ was set to 80 ms

**Fig. 9:**
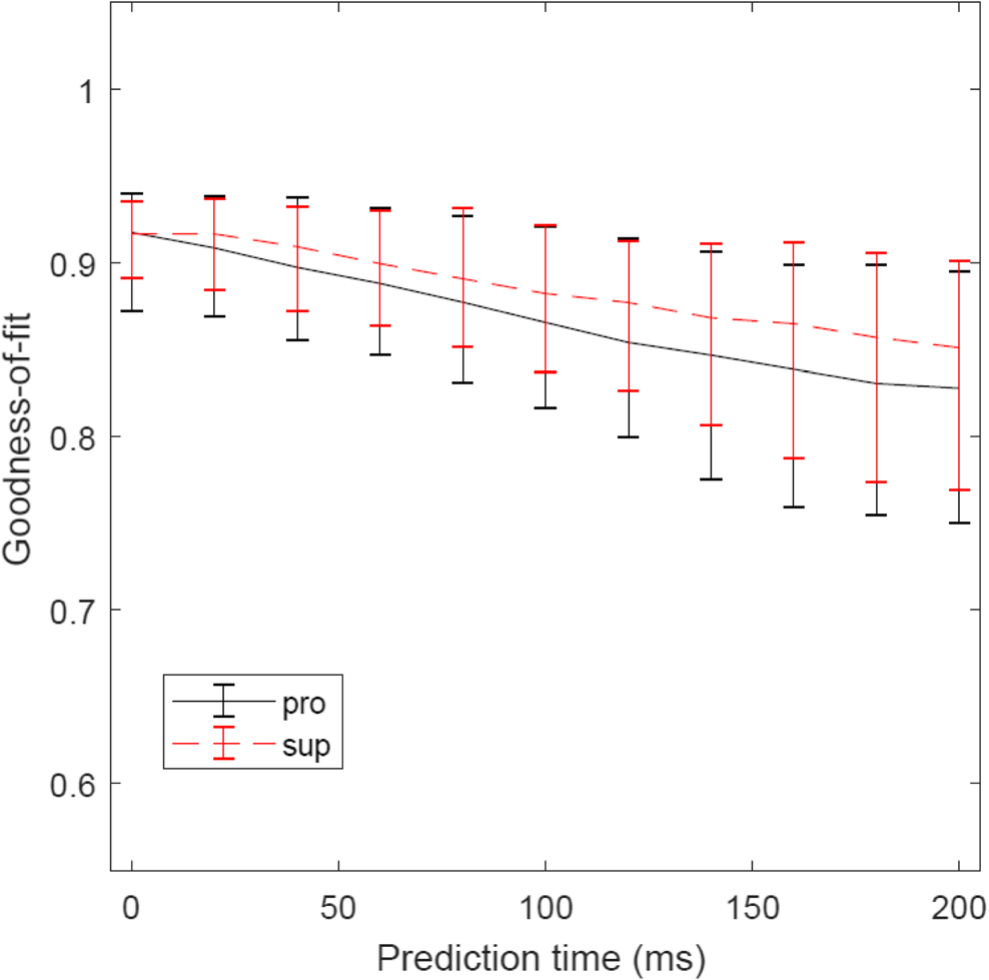
Goodness-of-fit of linear predictions with an increasing time advance *t*_1_ ranging from 0 to 200 ms with an interval of 20 ms. Error bars indicate standard deviations at each time advance. The goodness-of-fit was computed separately for the two postures: pronated (black solid line) and supinated (red dashed line)

Finally, we tested the forward-mode hypothesis of the cerebellum by predicting future inputs to the cerebellum (MFs) at time *t* + *t*_1_ from current outputs from the cerebellum (DCs) at time *t* (see “Linear Prediction of Future MF Activities from Current DC Activities”). The time advance *t*_1_ was varied from 20 to 200 ms in steps of 20 ms, and the reconstructions with *t*_1_ = 40 ms (Fig. 7) and *t*_1_ = 80 ms (Fig. 8) were presented. When *t*_1_ = 40 ms, spatiotemporal patterns of firing rates of MFs were captured by linear predictions without any noticeable delay. When *t*_1_ = 80 ms, although fitting to peaks and troughs becomes less accurate, the overall patterns of firing rates were preserved. In fact, the goodness-of-fit decreased moderately when *t*_1_ was increased from 20 to 200 ms (Fig. 9). One may suspect that any time series of similar complexity could predict the future input reasonably, so we proceeded on to test whether the performance of the linear prediction was a statistical change or not. Statistical significance of the goodness-of-fit was assessed by a bootstrap test based on a null hypothesis that any time series of similar complexity could predict the future MF activities. A bootstrap distribution of goodness-of-fit was constructed by shuffling the movement directions of DCs on the right-hand side of Eq. (13) for 10,000 times. Intuitively, DC activities with shuffled movement directions were of the same complexity of those with the experimental directions but did not retain movement-specific information. We found that the goodness-of-fit of the original data was significantly better than those of the shuffled data for *t*_1_ ranging from 20 to 100 ms for both the pronated (*p* < 5 × 10^−3^ for all *t*_1_ ranging from 20 to 100 ms, Bonferroni corrected) and supinated (*p* < 5 × 10^−3^ for all *t*_1_ ranging from 20 to 100 ms, Bonferroni corrected) postures. Therefore, the current output from the cerebellum contained predictive information about the future input to the cerebellum.

### Weight Distributions of Linear Reconstruction and Prediction Models

**Fig. 10:**
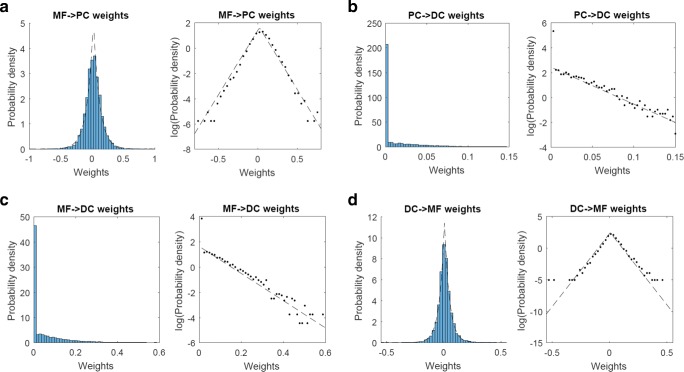
Distributions of weights of linear reconstruction models: **a** MF → PC connectivity, **b** PC → DC, and **c** MF → DC connectivity. Note that the PC → DC weights are nonpositive, and the MF → DC weights are nonnegative. The signs of PC → DC weights were flipped for a visual presentation. **d** DC → MF weights of linear prediction model trained with the time-advance parameter *t*_1_ = 40 ms. These distributions were normalized as probability density functions and were plotted in linear (left) and logarithmic (right) scales. Dashed lines indicate exponential distributions best fitted to the experimental distributions

Finally, we computed the distributions of weights of the linear reconstruction models of PCs (3) and DCs (5) and the linear prediction model of MFs (13). For the MF → PC connectivity, the weights exhibited an exponential distribution rather than a Gaussian (Fig. 10a), indicating that a relatively few MFs contributed dominantly to the reconstruction of each PC. For the MF → DC connectivity, most of the weights from MFs to DCs were zero reflecting the nonnegative constraint, and nonzero weights were distributed exponentially (Fig. 10b, left panel). Similarly, most of the weights from PCs to DCs were zero reflecting the nonpositive constraint, and nonzero weights were distributed exponentially (Fig. 10b, right panel). Therefore, relatively small number of MFs and PCs contributed to the reconstruction of firing rates of each DC. Finally, for the linear prediction model from DCs to MFs, the weights were again distributed exponentially (Fig. 10c), although there appeared outliers at large values of weights on both positive and negative ends. In summary, the weight distributions were all exponential but not Gaussian, indicating some structured projections between the separate populations in the cerebellar circuits.

Motivated by these exponential distributions of the weights, we further investigated the characteristics of the weights that enabled the linear reconstruction and prediction (see “Sparse Linear Analyses”). Specifically, we imposed explicitly sparseness on weights and performed sparse linear analyses that minimized the cost functions composed of a sum of squared error and a sparsity term as in Eqs. (14), (15), and (16). There were two findings. Approximately 60–80% of the input cells had nonzero weights, and about one third of input cells exhibited significantly large weights (Table 5). This analysis revealed that, within our dataset, only a few dozens of input cells contributed mainly to the linear reconstruction and prediction of a target cell.

**Table 5:** Summary of weight characteristics obtained in sparse linear analyses

	Proportion of nonzero weights	Proportion of significantly contributing weights
PC (pronated)	0.60 (0.14)	0.32 (0.082)
PC (supinated)	0.68 (0.13)	0.36 (0.096)
DC (pronated)	0.82 (0.34)	0.38 (0.26)
DC (supinated)	0.73 (0.354)	0.37 (0.14)
MF (pronated)	0.77 (0.10)	0.35 (0.063)
MF (supinated)	0.81 (0.12)	0.38 (0.067)

## Discussion

There are three main findings in this study. First, the distributions of firing rates of the three populations were all Gamma distributed, and they exhibited various degrees of spatiotemporal complexity. This indicated that the activities of the three populations represented functionally distinct roles in computation within the cerebellar circuit. Second, the firing rates of PCs are reconstructed linearly as a weighted sum of the firing rates of MFs, and the firing rates of DCs are reconstructed linearly as a weighted sum of the firing rates of MFs and PCs. Finally, the firing rates of DCs at time *t* linearly predict the firing rates of MFs at time *t* + *t*_1_, so the current output from the cerebellum contains predictive information about the future input to the cerebellum. These findings reveal the linear computation from one population to another and support the forward-model hypothesis of the cerebellum. It is worth mentioning that no nonlinearity that is expected from the perceptron model or dependence on previous inputs that is expected from the adaptive filter model was necessary to explain our data. Our results provide a strikingly simple picture of linear transformations for the cerebellar computation. In the following, we branch out to discuss our findings in consideration of previous studies and speculate implications for the computation of internal models in the cerebellum.

We should remark that all the results in this study were obtained from one animal and could have reflected an idiosyncrasy of that animal, so the conclusion in this study must be confirmed with another animal in a future study.

### Previous Electrophysiological Studies

There are two kinds of internal models hypothesized for motor control: a forward model that performs a state prediction from a current estimate and an efference copy, and an inverse model that transforms a desired goal of movement into the necessary motor commands [22]. There has long been a controversy over whether the cerebellum functions as a forward model or an inverse model. Previous single-unit recording studies of PC activities during hand movements provided controversial results for the internal-model hypothesis of the cerebellum [23, 24]. These studies examined the correlation between activities of PCs and movement kinematics and/or dynamics. The underlying assumption was that kinematic and dynamic representations of PCs relate to forward and inverse models, respectively. As kinematic variables (e.g., hand trajectory) and dynamic variables (e.g., muscle activities) are highly correlated under unperturbed conditions, one approach is to make a monkey perform the same movement trajectory with different loads on the hand to dissociate dynamics from kinematics ( [25, 26]. The monkey compensated the varying load in order to keep the same hand path, thereby dissociating the dynamics from the kinematics. For instance, Pasalar et al. [23] recorded simple-spike activities of task-related PCs while monkeys performed a circular manual tracking task under varying viscous and elastic loads. The simple-spike firing rates and spatial tuning did not change significantly under various load conditions, which supported a kinematic representation of arm movements in the cerebellar cortex. Their results appeared to be compatible with the forward-model hypothesis of the cerebellum, which predicts movement kinematics.

Similarly, Yamamoto et al. [24] recorded simple-spike activities of PCs while monkeys performed elbow extension or flexion movements under assistive or resistive forces. In contrast with the findings of Pasalar et al., the simple-spike activities did change according to the load condition and correlated with the change in muscle activities, thereby seemingly consistent with the inverse-model hypothesis. Although the two studies examined PC firing rates in similar experiments, their conclusions were opposite to each other. Other studies described simple-spike activities correlated with eye-movement dynamics [27] or cursor-movement kinematics [28]. Therefore, to date, these single-unit–recording studies seem inconclusive about whether the cerebellum plays a role of an internal forward model or inverse model.

These studies rely on an assumption that kinematic and dynamic representations of PCs relate to forward and inverse models, respectively. This assumption, however, does not hold because an internal forward model should include dynamical variables such as efference copies of motor control signals. Also, disentangling predicted state signals, sensory feedback signals from the periphery, and motor commands is rather difficult because these signals resemble each other [8]. Therefore, a mere correlative comparison of PC activities with one or other behavioral parameters would not lead to conclusive evidence for either of forward or inverse models.

### State Prediction as a Prerequisite for a Forward Model

To resolve the limitation of the single-unit studies that correlated firing rates of one cell population and behavioral measures, we believe it essential to analyze network-level computation across multiple cell populations, as suggested by Wolpert and Miall [8]. The current study was designed to circumvent the abovementioned difficulty of disentangling multiple representations and targeted the transformation through the cerebellum from MF (input to the cerebellum) to DC (output from the cerebellum) via PC (output from the cerebellar cortex to the cerebellar nuclei), revealing the linear computation from one cell population to another. Furthermore, we found that the current output of the cerebellum predicts the future inputs to the cerebellum, a distinctive feature of an internal forward model. These findings could not have been achieved with the analysis of correlation between activities of one cell population and behavioral measures.

A critical test of the forward-model hypothesis of the cerebellum is whether the prediction performed in the cerebellum can offset delayed sensory feedback. Delays in sensory feedback can differ from one sensory modality to another; proprioceptive feedback takes of the order of 50 ms from muscle spindles to the somatosensory and motor cortices, and visual feedback takes about 50 ms from the retina to the primary visual cortex and 100 ms to the higher visual cortices [29]. These delays can deteriorate the performance of rapid movements of the order of a few hundred milliseconds employed in this study. Our analysis revealed that the current DC activity contained predictive information about the future MF activity for a range of time advance. Therefore, our result supports that the cerebellum is capable of compensating the sensory delays of the order of 100 ms, supporting the forward-model hypothesis. A previous electrophysiological study reported that activity of postcentral neurons changed on the average of about 60 ms before the onset of agonist elbow muscles in voluntary elbow movements [30]. The early onset of activity of postcentral neurons is within the timescale of prediction in the cerebellum found in this study.

Morphological and physiological evidence accumulated over decades suggests that a region of the cerebro-cerebellum that forms a closed-loop circuit with M1 appears to satisfy the basic requirements for a forward model that generates a prediction of the outcome of a motor command [31]. First, this region of the cerebro-cerebellum receives a putative efference copy as well as a strong somatosensory input [14, 15], and these inputs are presumed to be integrated in the cerebellar cortex. Second, the activities of PCs in this region lag behind those of M1 neurons, while they precede the movement onset [15]. The timing of activity is compatible with the idea that it works as a forward model that predicts an outcome of the motor command. As a result, the output of this region of the cerebro-cerebellum may help M1 to generate a suitable motor command for the next moment depending on the predicted consequence before a feedback signal is available for the current motor command. We note that there are in general two input pathways to the MFs: one from the cerebral cortex through the pons and another from the peripheral sensory organs. Our single-unit recording did not allow to identify the origin of MFs and thus to discuss what information the MF activities encoded.

Our analyses assume the feedforward anatomical structure of the cerebellar circuit, but it is known that the cerebellar circuit contains recurrent anatomical connections that form a closed-loop circuit within the cerebellum, such as those composed of Golgi and granule cells and those composed of Purkinje and basket cells. Among these recurrent connections, the most relevant to this study is the nucleocortical projections from the cerebellar nuclei to the granular layer as MFs [32]. A recent study reported that excitatory output cells in the interposed nucleus provide efference copy signals via MFs to the cerebellar cortical zones and that an eye-blink conditioning training increased the local density of nucleocortical MF terminals [33]. One may suspect, therefore, that the linear prediction from DCs to MFs reported in this study could be attributed to the nucleocortical recurrent connections. We note, however, that this nucleocortical projection per se does not explain the longer time scale of linear prediction up to 100 ms reported in this study. Also, the nucleocortical pathway comprises only approximately 5% of the total of cerebellar MF inputs [34]. We hence expect that recurrent connections in the nucleocortical projections have minor contributions to our results of linear prediction from DCs to MFs.

### Linear Computation in the Cerebellar Circuit

We have shown the linear transformations from MFs to PCs and from MFs and PCs to DCs explained the observed firing rates recorded during the wrist movement task. In addition, the future MF activities were linearly predicted from the current DC activities. The success of linear modeling of PC and DC activities was unexpected and intriguing for the following three reasons. First, a PC receives parallel fiber inputs of the order of 100,000, while our dataset from monkey 1 contained only 94 MFs. Second, these firing rates were recorded at different sessions or even across different recording days separated by years, so the firing rates of the three populations were of no direct causal relation. Nonetheless, the computation in the cerebellar circuit turned out to be linear.

A missing piece in our study is the granule cell activity. Our results demonstrated that the transformation from MFs to PCs is linear, implying another linear computation in the granular layer. Two possibilities of computation in the granular layer are suggested. The first possibility is that each granule cell performs linear computation by linearly summing up the inputs from MFs. A previous study reported linear computation from MFs to medium ganglion cells in the cerebellum-like structure of electric fish [35]. They reported that linear weighted sums of sparsely and randomly mixed MF inputs reconstructed the membrane potentials of granule cells. The reconstructed granule cell activities exhibited a rich repertoire of temporal bases, which in turn constitute a negative image of sensory inputs. Their study suggests the linear computation from MFs to granule cells, in line with our results. It is interesting to note that, in their study, sparsity of linear weights was explicitly incorporated into an error function. In contrast, our results demonstrated that the sparse distributions of weights emerged spontaneously without a sparseness term in the cost function. Another possibility is that each granule cell performs nonlinear computation of MF inputs and the population of granule cells as a whole encodes inputs linearly. Recent studies demonstrated that individual granule cells were more narrowly tuned to the whisker angle of a rat than EPSC, thereby exhibiting nonlinear computation of granule cells sharpening their inputs [36], while individual PCs encoded whisker position linearly [37]. Interestingly, the population of narrowly tuned granule cells provides a linear excitatory drive across a range of whisker positions to PCs [36]. Because our dataset does not contain granule cell activities, we are not certain which may be the case.

Despite the fact that there are interneurons with recurrent connections in the cerebellar circuit, our finding indicates that the computation in the cerebellum is unexpectedly linear. We here speculate two possible explanations for the success of our linear modeling. One reason is that the performance of the monkeys was stable because they have been trained over years for this wrist movement task. Therefore, we expect that the response properties of cerebellar cells remained stable across experimental sessions once the monkeys had achieved stable task performance. Another reason is that the cells in the dataset were selected if they showed task-related modulations of firing rates. Among numerous parallel-fiber inputs to a PC, it is conceivable that only a fraction of task-related inputs determines the response properties of that PC, as revealed by the sparse linear analyses. The stability of task performance and the selected sampling of task-related cells could explain the success of our linear modeling.

In line with our findings of linear transformations and predictions, an increasing number of recent literatures have reported that firing rates of cerebellar cells encode movement-related parameters linearly [38]. In the case of MFs [39, 40], Laurens et al., for example, reported a linear monotonic relationship between firing rate of MFs and eye position [39]. Similarly, for PCs [27, 37, 41–47], Hong et al. found that regularly firing spikes perform linear encoding of eye movement velocity by firing rate [43]. Finally, neurons in the cerebellar nuclei [48–51], the timing, and kinematics of motor output were modulated by linearly graded disinhibition of neurons in the deep cerebellar nuclei [49]. Along with these reports of linear encoding of behavioral parameters in the cerebellar cells, our findings reinforce the perspective of linear computation within the cerebellar circuits. Whereas there is evidence for linear encoding in the cerebellum as referenced above, we note that there is also evidence for nonlinear coding of saccade onset timings by spikes of PCs that are related to the period of occasional pauses [43], suggesting a possibility of multiplexed encoding by cerebellar cells.

### Linear Equations and Interpretation as Kalman Filter

**Fig. 11:**
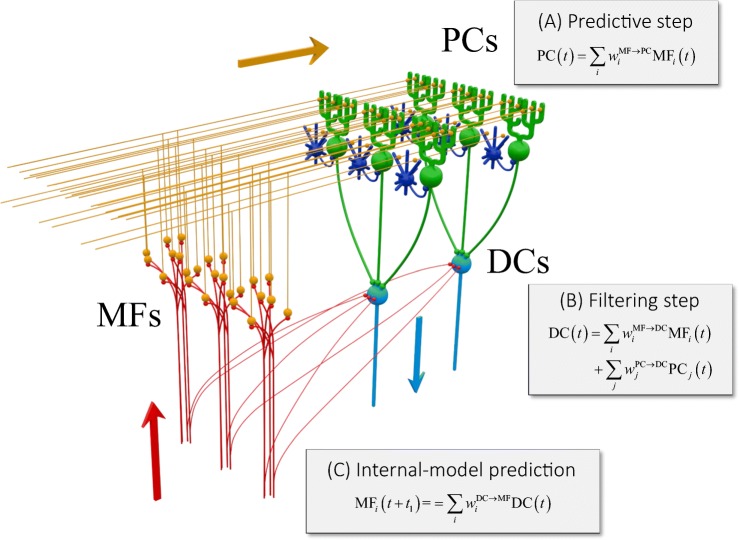
Summary schematic of our findings overlaid on the cerebellar circuit. MF, mossy fiber (red); PC, Purkinje cell (green); DC, dentate cell (light blue). Granule cells (orange) and inhibitory interneurons (blue) that are not analyzed in this work are included to show the basic structure of the cerebellar neuron circuitry. Three stages of linear computation obtained in our analysis are accompanied with the three types of computation of Kalman filter explained in the main text

The three linear equations derived from the firing rates are summarized as follows,

17 $$ {\displaystyle \begin{array}{l}{\widehat{\mathrm{PC}}}_i(t)=\sum \limits_{j=1}^{N_{\mathrm{MF}}}{w}_{ij}^{\mathrm{MF}\to \mathrm{PC}}{\mathrm{MF}}_j(t)\\ {}{\widehat{\mathrm{DC}}}_i(t)=\sum \limits_{j=1}^{N_{\mathrm{MF}}}{w}_{ij}^{\mathrm{MF}\to \mathrm{DC}}{\mathrm{MF}}_j(t)+\sum \limits_{k=1}^{N_{\mathrm{PC}}}{w}_{ik}^{\mathrm{PC}\to \mathrm{DC}}{\mathrm{PC}}_k(t)\\ {}{\widehat{\mathrm{MF}}}_i\left(t+{t}_1\right)=\sum \limits_{j=1}^{N_{\mathrm{DC}}}{w}_{ij}^{\mathrm{DC}\to \mathrm{MF}}{\mathrm{DC}}_j(t)\end{array}} $$

and as in the schematics (Fig. 11). The activities of PCs are linear summations of activities of MFs, and the activities of DCs are linear summations of activities of MFs and PCs. There are two functional projections from MFs in the linear equations: one from MFs to PCs and the other from MFs and DCs. The two projections demonstrated little overlap, indicating that these two projections might convey functionally distinct information.

We realized that the chain of linear equations of neuron activities resembles those of an estimator known as Kalman filter. If we assume that MFs represent a current estimate of state and sensory feedback, PCs represent a prediction of state, and DCs represent a filtered state, then the linear equations can be interpreted as Kalman filter as follows. The first equation is a prediction step ((A) in Fig. 11); a current estimate (MFs) is projected to a predicted state (PCs). Then the second equation is a filtering step; a predicted state (PCs) and a sensory feedback (MFs) are integrated into a filtered state (DCs) ((B) in Fig. 11). Then, a filtered state (DC) contained predictive information about the future inputs (MFs) ((C) in Fig. 11).

In the above analogy with the Kalman filter, we have made an important assumption: PCs and DCs receive information about current estimate of state and sensory afferent signals through MFs, respectively. Our statistical test suggests that MF projections to PCs differ from MF collateral projections to DC in terms of their sources. It is known for the cerebro-cerebellum that MFs originate from the pons (which receives direct cortical projections) and from the brainstem/spinal cord, and that each of the MF inputs reflects either cortical activities or sensory feedback signals. Anatomical studies using an anterograde tracer (HRP-WGA) in cats reported that MFs of cortical origins via pons consist of the main input to the cerebellar cortex, whereas collaterals of those MFs poorly project directly to the dentate nuclei [52, 53]. Therefore, “the bulk of information of cortical origin reaches the cerebellar nuclei only after processing in the cerebellar cortex” (p. 22 of Brodal et al. [52]). This is consistent with our assumption that DCs receive a predicted state not from MFs but from PCs.

One critical aspect of Kalman filter is the Kalman gain which balances a predicted state and an observed state in an optimal way. In Kalman filter, a filtered state ${\widehat{\mathbf{x}}}_{t\mid t}$ combines a predicted state ${\widehat{\mathbf{x}}}_{t\mid t-1}$ and an observed state **z**_*t*_ as in

18 $$ {\widehat{\mathbf{x}}}_{t\mid t}={\widehat{\mathbf{x}}}_{t\mid t-1}+\mathbf{K}\left({\mathbf{z}}_t-\mathbf{C}{\widehat{\mathbf{x}}}_{t\mid t-1}\right)=\left(\mathbf{I}-\mathbf{KC}\right){\widehat{\mathbf{x}}}_{t\mid t-1}+\mathbf{K}{\mathbf{z}}_t $$

Here **K** is the Kalman gain and **C** is the observation matrix. By comparing the second equation of (17) and Eq. (18), we speculate that the weights from PC to DC (*w*^PC → DC^) and the weights from MF to DC (*w*^MF → DC^) correspond to the matrices **I** − **KC** and **K** in Eq. (18), respectively. While these weights were assumed to be stable and constant in our analysis because the task performance of the monkey was unchanged for years, the analogy predicts an opposing plasticity of *w*^PC → DC^ and *w*^MF → DC^, namely, *w*^PC → DC^ and *w*^MF → DC^ should change their strengths in opposite directions when learning occurs. Although the analogy between the cerebellum and Kalman filter presented here is a speculation, we believe that this analogy could serve as a computational proposal that drives future studies of the cerebellum.

### Previous Computational Models of the Cerebellum

There are lines of computational models of the cerebellum in the literatures. The pioneering and most dominant model of the cerebellum is the perceptron model of the cerebellar cortex by Marr [54] and independently by Albus [55]. The perceptron model was first inspired by the analogy of feedforward network structures between the cerebellar cortex and perceptron. The core hypothesis was that two independent inputs to a PC (MFs and a climbing fiber) represent input pattern signals and supervised error signals, respectively. Later, the climbing fiber inputs were found to induce long-term depression in synapses between parallel fibers and a PC in rabbits’ cerebellar slices [56, 57]. The perceptron model contains a nonlinear term to threshold a weighted sum of inputs. On the contrary, our results have shown that the linear model sufficed to explain the firing rates of PCs in terms of MFs.

The perceptron model is essentially a static pattern classifier and not designed to handle time-varying, dynamic inputs. Subsequently, the perceptron model was extended to the adaptive filter model which generates a dynamic response by summing various temporal basis patterns [58]. The adaptive filter model considers a recurrent circuit among MFs, Golgi cells, and granule cells which generates resonant temporal patterns with various phase leads and lags. Therefore, the adaptive filter model assumes that the activities of PCs result from the interaction among MFs, Golgi cells, and granule cells. There is supportive evidence of the adaptive filter model [59, 60]. On the contrary, our finding of linear transformation from MFs to PCs suggests that the recurrent circuit plays a negligible role in generating temporal bases and rather that MFs already contain rich temporal repertoire that in turn drives the activities of PCs. The present study cannot exclude a possibility that different parts of the cerebellum may adopt different neural mechanisms for generating temporal patterns; the adaptive filter model has been tested in the floccus, whereas our data was recorded from the cerebellar hemisphere.
